# Effect of *Artocarpus communis* Extract on UVB Irradiation-Induced Oxidative Stress and Inflammation in Hairless Mice

**DOI:** 10.3390/ijms14023860

**Published:** 2013-02-12

**Authors:** Chiang-Wen Lee, Horng-Huey Ko, Chee-Yin Chai, Wan-Tzu Chen, Chun-Ching Lin, Feng-Lin Yen

**Affiliations:** 1 Department of Nursing, Division of Basic Medical Sciences, Chronic Diseases and Health Promotion Research Center, Chang Gung Institute of Technology, Chia-Yi, Taiwan; E-Mail: cwlee@gw.cgust.edu.tw; 2 Department of Fragrance and Cosmetic Science, College of Pharmacy, Kaohsiung Medical University, Kaohsiung, Taiwan; E-Mail: hhko@kmu.edu.tw; 3 Department of Pathology, Kaohsiung Medical University Hospital, Kaohsiung, Taiwan; E-Mails: cychai@kmu.edu.tw (C.-Y.C.); wanzi@kmu.edu.tw (W.-T.C.); 4 School of Pharmacy, College of Pharmacy, Kaohsiung Medical University, Kaohsiung, Taiwan; E-Mail: aalin@kmu.edu.tw

**Keywords:** *Artocarpus communis*, ultraviolet, antioxidant, anti-inflammation, photoprotective

## Abstract

Administration of antioxidants and anti-inflammatory agents is an effective strategy for preventing ultraviolet (UV) irradiation-induced skin damage. *Artocarpus communis* possesses several pharmacological activities, such as antioxidant, anticancer and anti-inflammation. However, the photoprotective activity of methanol extract of *A. communis* heartwood (ACM) in ultraviolet irradiation-induced skin damage has not yet been investigated. The present study was performed using ultraviolet absorption, histopathological observation, antioxidant and anti-inflammation assays to elucidate the mechanism of the photoprotective activity of ACM. Our results indicated that ACM displayed a UVA and UVB absorption effect and then effectively decreased scaly skin, epidermis thickness and sunburn cells during ultraviolet irradiation in hairless mice. ACM not only decreased ultraviolet irradiation-mediated oxidative stress, including lowering the overproduction of reactive oxygen species and lipid peroxidation (*p* < 0.05), but also reduced the levels of pro-inflammatory cytokines, including tumor necrosis factor-α (TNF-α) and interleukin 1β. Additionally, ACM can decrease the synthesis of cytosolic phospholipase A2, cyclooxygenase, inducible nitric oxide synthase and vascular cell adhesion molecular-1 via inhibiting TNF-α-independent pathways (*p* < 0.05) in UVB-mediated inflammation and formation of sunburn cells. Consequently, we concluded that ACM extract has a photoprotective effect against UVB-induced oxidative stress and inflammation due to its sunscreen property, and its topical formulations may be developed as therapeutic and/or cosmetic products in further studies.

## 1. Introduction

Overexposure of the skin to ultraviolet (UV) radiation has been shown to induce several pathological changes in skin damage, including erythema, scaling, edema, hyperpigmentation, skin thickness, sunburn cells (apoptotic keratinocyte), immunosuppression, tumorigenesis and aging [1–4]. During chronic UV radiation, excessive reactive oxygen species (ROS), such as superoxide anion (·O_2_^−^), hydroxyl radical (·OH), hydrogen peroxide (H_2_O_2_) and singlet oxygen (^1^O_2_), can break the balance between prooxidant production and antioxidant defense and, therefore, directly promote oxidative DNA damage and peroxidation of lipid and protein in the skin [5] and, finally, cause the related oxidative stress diseases, including photoallergy [6], photoaging [7] and photocarcinogenesis [8]. Overproduction of ROS is also an inducible factor caused by the overexpression of pro-inflammatory cytokine during chronic UVB irradiation, such as tumor necrosis factor-α (TNF-α) and interleukin 1, which triggered the expression of cytosolic phospholipase A2 (cPLA2) and cyclooxygenase (COX-2), therefore leading to an increase of the prostaglandin level, the formation of sunburn cells and inflammatory cell infiltration and activation [9–11]. In addition, TNF-α also activates the expression of inducible nitric oxide synthase (iNOS) and vascular cell adhesion molecular-1 (VCAM-1) in UVB-induced skin damaged hairless mice, leading to inflammation progression [12,13].

Thus, antioxidant supplements have been shown to attenuate inflammation and cell death through decreasing oxidative stress, such as traditional medicines [14] and natural products [15]. Previous studies have demonstrated that polyphenols act as powerful antioxidants to prevent UV-induced oxidative stress and inflammation, such as anthocyanins [16] and quercetin [17]. *Artocarpus communis*, mainly grown in Taiwan and Southeast Asia, is extensively used in food, traditional medicine and the agricultural industry. The main active components of *A. communis* are flavonoids (artocarpine, norartocarpetin), chalcones (isobacachalcone, morachalcone A, gemichalcone B) and stilbenes (oxyresveratrol and chlorophorin) [18,19]. *A. communis* has been reported to possess many pharmacological activities, including antityrosinase [20], 5α-reductase inhibitor [21] and anticancer [22]. However, there has been no study on the effect of the methanol extract of *A. communis* on UVB irradiation-induced photodamage *in vivo*.

The aim of the present study was to investigate the photoprotective effects of the methanol extract of *A. communis* on UVB-induced photodamage in a hairless mice model. In this study, we performed the morphological and histopathological examinations, antioxidant activity and anti-inflammation assays to evaluate the photoprotective effects of *A. communis* in UVB-induced skin damaged hairless mice.

## 2. Results and Discussion

It is well known that chronic UV irradiation can induce skin damage with a number of pathological changes, including DNA damage [1,2], formation of sunburn cells (apoptotic keratinocyte) [23], oxidative stress induction by ROS overproduction [8] and over release of proinflammatory cytokines [24]. However, botanical administration is a good strategy to protect the UV radiation-induced pathological changes of skin in recent years, such as green tea polyphenols [25] and *Scutellaria baicalensis* [26]. In the present study, the methanol extract of *A. communis* was observed to exhibit a photoprotective effect, as demonstrated by decreasing epidermis thickness and formation of sunburn cells in hairless mice induced with UVB irradiation. Moreover, ACM diminished the amount of ROS and lipid peroxidation against UVB-induced skin damage. ACM also decreased the release of proinflammatory cytokines, TNF-α and IL-1β, and inflammatory protein expression, COX-2, cPLA2, iNOS and VCAM-1. Accordingly, we suggest that the reduction of oxidative stress and inflammation have played the major role in the mechanism of photoprotective effects.

### 2.1. UV-Absorption Properties of ACM

**Figure 1:**
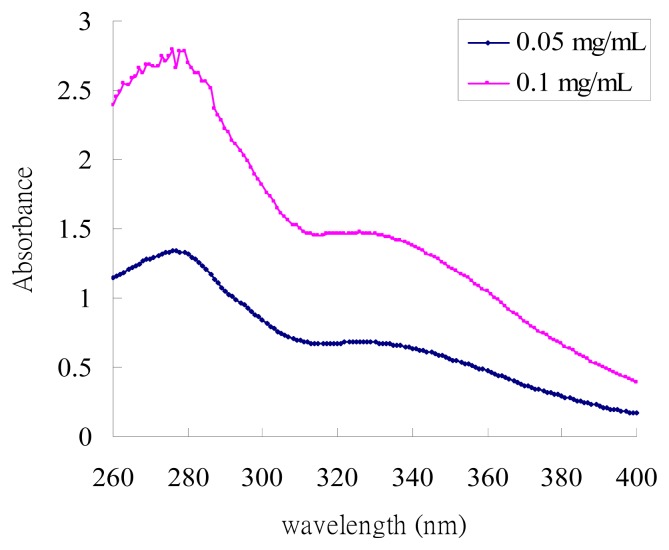
UV absorption spectrum (260–400 nm) of *Artocarpus communis* methanol extract.

As shown in Figure 1, the UV-absorption spectra of ACM displayed the highest absorption in the UVB field (*λ*_max_ = 279 nm) and the second one in the UVA field (*λ*_max_ = 326 nm). These results indicated that ACM possessed good absorption of UVA and UVB radiation and, therefore, presented a good sunscreen effect.

### 2.2. Effects of ACM on the Skin Morphology and Histopathological Changes in UVB-Induced Skin Damage

**Figure 2:**
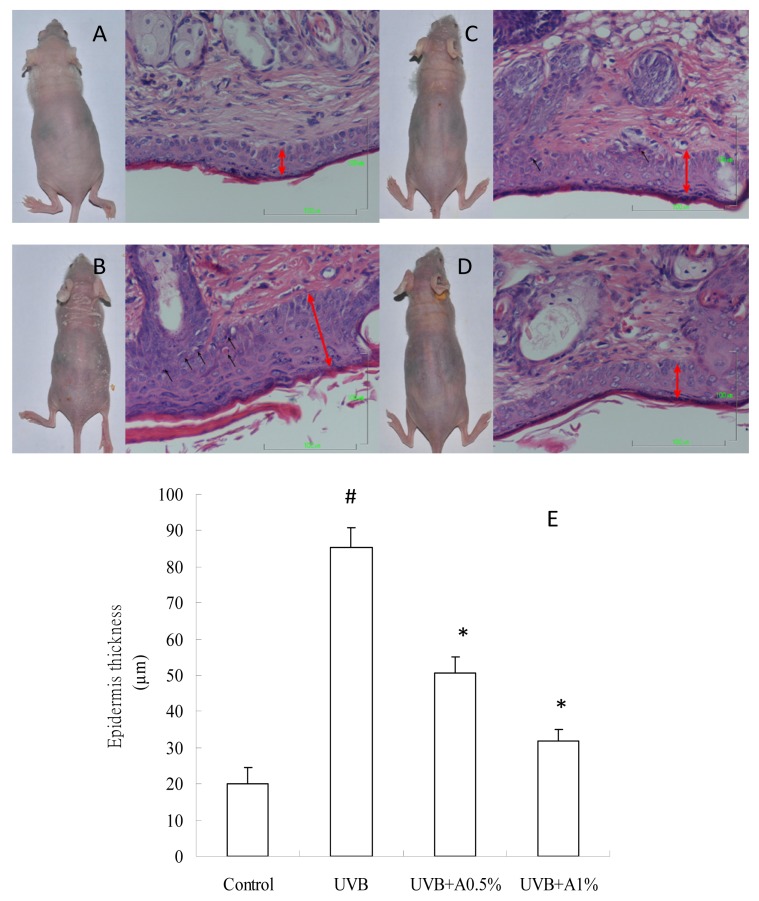
*Artocarpus communis* methanol extract attenuated skin scaling, epidermis thickness and the formation of sunburn cells in UVB-irradiated hairless mice. (**A**) control; (**B**) UVB irradiation; (**C**) pretreatment with ACM 0.5% after UVB irradiation; (**D**) pretreatment with ACM 1% after UVB irradiation; (**E**) the result of epidermis thickness on different groups is represented by the mean ± SD (*n* = 4). Statistical significance was defined as *p* < 0.05.

The results of morphology and histopathological changes on the skin tissues of various groups are shown in Figure 2. Obvious scaly skin was found in the UVB-induced group when compared to the control group. However, 0.5% of ACM treatment can reduce UVB-induced skin damage, but, still, a few presented with scaly skin, and there is no obvious scaly skin present in 1% of the ACM treatment. In addition, sunburn cell (apoptotic keratinocyte) is a major index to evaluate skin damage after UVB irradiation. The black arrow in Figure 2 indicates that the formation of sunburn cells was obviously presented in UVB-induced skin damaged hairless mice (Figure 2B), but not found in the vehicle group (Figure 2A). However, there is a few sunburn cells found in 0.5% of the ACM treatment (Figure 2C), and the observation of the formation of sunburn cells did not manifest in topical administration with 1% of ACM treatments (Figure 2D). Moreover, the red arrow in Figure 2 indicates the epidermis thickness in hairless mice. The epidermis thickness in the UVB irradiation group (Figure 2B) is obviously increased when compared to the vehicle group (Figure 2A), and ACM treatments effectively attenuated the UVB irradiation (Figure 2C,D). Furthermore, Figure 1E shows that the epidermis thickness of the UVB-induced group (85.41 ± 5.31 μm) increased approximately by 4.2-fold compared to the control group (20.20 ± 4.45 μm) (*p* < 0.05). Zero-point-five-percent (50.63 ± 4.53 μm) and 1% (31.76 ± 3.26 μm) of ACM were significantly lower in epidermis thickness in the UVB-induced skin damage hairless mice when compared with the vehicle group (*p* < 0.05). Our result also indicated that 1% of ACM exerts a better photoprotective effect than 0.5%. These results indicated that ACM is a good photoprotective agent to prevent UVB-induced skin damage. It was reported that overexposure of skin to UV irradiation induced an increase of skin scaling and epidermis thickness, resulting in hyperkeratinization and hyperplasia in human and hairless mice [27,28]; furthermore, chronic UV irradiation is a complete carcinogen to trigger both initiation and progression of skin cancer [29]. Therefore, administration of photoprotective supplements can be used against UV irradiation-induced skin scaling and histopathological changes, such as quercetin [17] and soy isoflavones. In the present study, epidermis thickening is considered one of the main markers of UVB irradiation damage for confirming the photoprotective effect of natural products. Our results demonstrated that topical administrations of ACM (0.5% and 1%) not only reduced irradiation, with UVB light results in the scaly skin, but also decreased the epidermis thickness and the formation of sunburn cells. Therefore, ACM can be used as a photoprotective agent to prevent photodamage after UVB irradiation.

### 2.3. Antioxidant Activity of ACM on UVB-Induced Oxidative Stress

**Figure 3:**
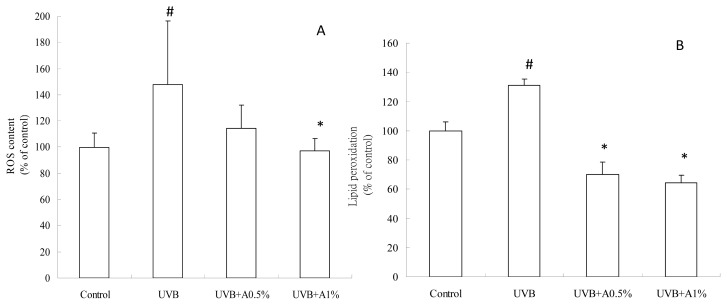
The index of the oxidative stress of skin tissue in UVB-irradiated skin damage in hairless mice: (**A**) ROS content; and (**B**) lipid peroxidation. Values are expressed as the mean ± SD, *n* = 4; # *p* < 0.05 *vs*. control group; ^*^p < 0.05 *vs*. UVB-irradiated group.

It is well known that UVB irradiation can generate excessive ROS production and cause oxidative DNA damage, resulting in the formation of oxidative stress. At the same time, excessive ROS rapidly initiate the peroxidation of the unsaturated fatty acids of phospholipids and protein in the keratinocyte membrane, leading to cell death. However, a great number of studies have suggested the photoprotective effect of topical antioxidants, which might be a successful strategy for reducing UV irradiation-induced oxidative damage of the skin, such as *Curcuma longa* [15] and phenolic acids [30]. In the present study, ROS overproduction and lipid peroxidation are the oxidative stresses that cause skin injury in UVB irradiation-induced photodamage hairless mice. Our results demonstrated that topical administration of ACM effectively decreased the amount of ROS and then reduced lipid peroxidation. Therefore, we suggest that ACM displays an antioxidant role in the photoprotective mechanism by reducing oxidative stress. Figure 3 shows the change of ROS content and lipid peroxidation from the control, pretreatment with vehicle and the 0.5% and 1% of ACM groups. The ROS content of vehicle treatment with the UVB-induced group was increased (Figure 3A). Pretreatment with 0.5% and 1% of ACM significantly lowered the ROS content of skin tissue (*p* < 0.05). Additionally, it is well known that ROS overproduction triggered a chain reaction on lipids and led to cell death. Figure 3B show that 0.5% and 1% of ACM treatment significantly reduced the lipid peroxidation of skin tissue when compared to the vehicle group with UVB irradiation (*p* < 0.05). These results indicate that ACM had an antioxidant activity to protect against UVB-induced oxidative stress.

### 2.4. Anti-Inflammatory Activity of ACM on Cytokine Levels in UVB-Induced Skin Inflammation

**Figure 4:**
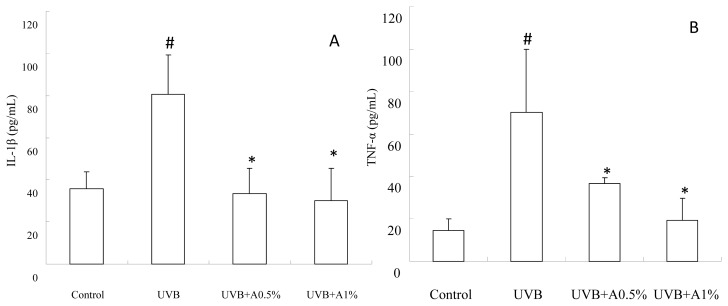
The pro-inflammatory cytokines of skin tissue on UVB-irradiated skin damage in hairless mice: (**A**) TNF-α; and (**B**) IL-1β. Values are expressed as mean ± SD, *n* = 4; # *p* < 0.05 *vs.* control group; ^*^p < 0.05 *vs.* UVB-irradiated group.

**Figure 5:**
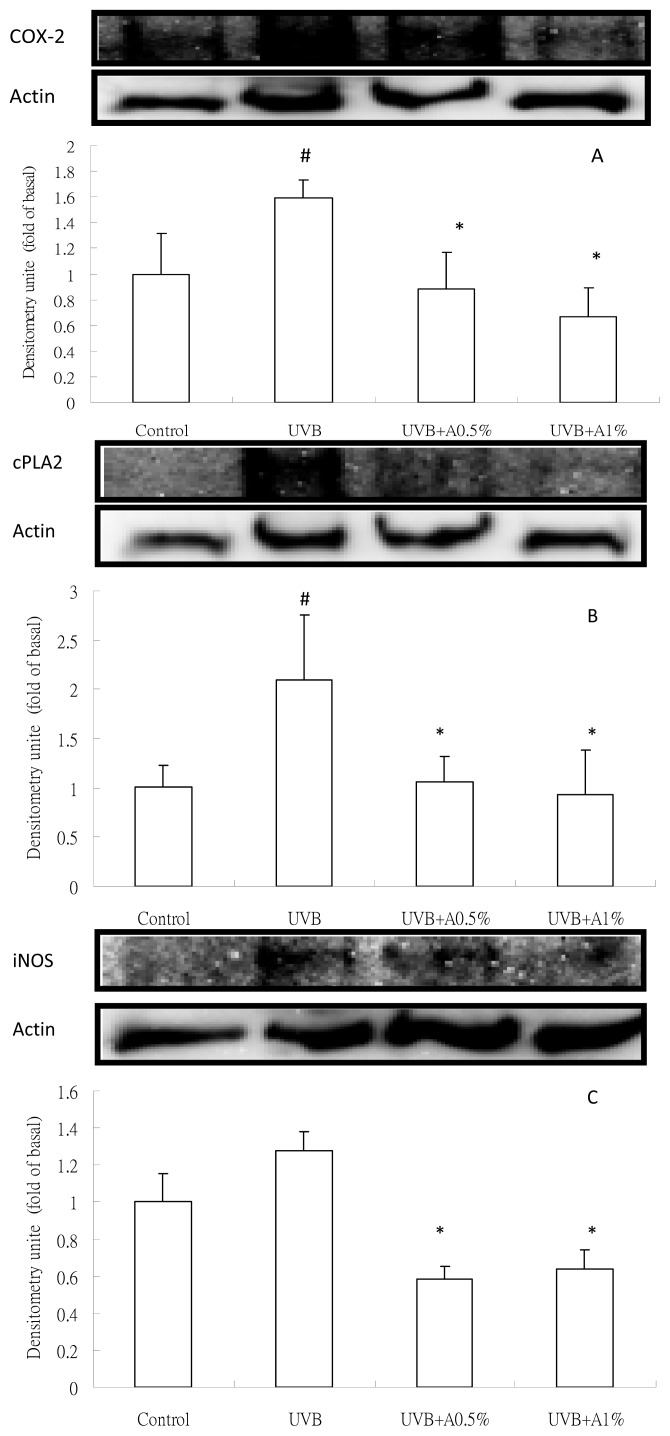
Inflammatory protein expression of skin tissue on UVB-irradiated skin damage in hairless mice: (**A**) cPLA2; (**B**) COX-2; (**C**) iNOS and VCAM-1. # *p* < 0.05 *vs.* control group; ^*^p < 0.05 *vs.* UVB-irradiated group.

As shown in Figure 4, UVB-irradiation can cause a significant increase in TNF-α and IL-1β levels when compared with the vehicle group (*p* < 0.05). However, UVB-induced skin damaged mice treated with 0.5% and 1% of ACM significantly reduced the TNF-α (Figure 4A) and IL-1β (Figure 4B) levels in the skin homogenate as compared with the UVB-induced-intoxicated group (*p* < 0.05). 1% of ACM treatment has a better effect on TNF-α levels as compared with 0.5% of ACM (*p* < 0.05), but a significant decrease did not present itself on IL-1β levels. In addition, the inflammatory protein expression after UVB irradiation is shown in Figure 5. There was a significant increase of COX-2, cPLA2, iNOS and VCAM-1 protein levels in the UVB-irradiation treated vehicle group, approximately by 1.6-fold, 2.1-fold, 1.3-fold and 2.3-fold, respectively, compared to the control group (*p* < 0.05). 0.5% and 1% of ACM treatments had a significant reduction of COX-2 (Figure 5A), cPLA2 (Figure 5B), iNOS (Figure 5C) and VCAM-1 (Figure 5D) protein in UVB-induced skin inflammation (*p* < 0.05).

Previous studies have demonstrated that ROS overproduction also plays an important role in the UVB irradiation-induced immediate inflammatory response in the skin, including erythema, edema and leukocyte infiltration. ROS can trigger the transcription and release from skin keratinocytes of pro-inflammatory cytokines, including TNF-α and IL-1β [31,32]. Previous studies have also demonstrated that these proinflammatory cytokines appear to be involved in the formation of sunburn cells (keratinocyte apoptosis) and, then, increase the levels of metalloproteases and degrade the dermal collage and elastic fibers during UVB irradiation-induced skin damage [33–35]. In the present study, our results demonstrated that three weeks of UVB irradiation significantly increased the levels of TNF-α and IL-1β in skin homogenates, and sunburn cells were also found in our histopathological observations (Figure 2). However, topical administrations of ACM (0.5% and 1%) effectively reduced the release of TNF-α and IL-1β from skin cells and also decreased the formation of sunburn cells. On the other hand, a great number of studies suggest that the overexpression of TNF-α can participate in the synthesis of COX-2, cPLA2 and iNOS in UVB irradiation-mediated inflammation [10,16,36]. Chen *et al*. have also indicated that UVB irradiation increased cPLA2 synthesis and plays a significant inflammation role by enhancing prostaglandin production in UVB irradiation-induced skin damage. UVB irradiation-mediated cPLA2 induction may metabolize phospholipids to arachidonic acid. immediately causing membrane lipid peroxidation, leading to skin cell death [10]. In addition, Isoherranen *et al*. have indicated that enhanced COX-2 expression has another important role for increasing prostaglandin synthesis and inflammation response during UVB irradiation [11]. Sethi and Sodhi also demonstrated that iNOS can overproduce the amount of nitric oxide for triggering the inflammation pathway, which can promote the formation of apoptotic keratinocyte and progression of skin carcinogenesis [37]. Our data indicated that UVB irradiation-mediated oxidative stress and TNF-α increase not only induced cPLA2 expression, but also increased COX-2 and iNOS. However, ACM is able to decrease the expressions of cPLA2, COX-2 and iNOS by reducing the chain reaction of inflammation and, thereby, preventing skin cell apoptosis from the overproduction of ROS and TNF-α. Moreover, Jones *et al*. demonstrated that the cell adhesion molecule, VCAM-1, is expressed in normal skin tissue, but overexpressed in autoimmune connective tissue disease, such as systemic sclerosis and lupus erythematosus [36]. Our results found significantly greater expression of VCAM-1 in skin tissue in UVB irradiation hairless mice compared with the vehicle group. However, ACM treatment (0.5% and 1%) can decrease the expression of VCAM-1 after UVB irradiation. This indicated that ACM can attenuate the VCAM-1-mediated cell-matrix inflammation by preventing UVB-induced skin damage. Therefore, we suggested that ACM displays another role in the photoprotective mechanism by preventing inflammatory response.

## 3. Experimental Section

### 3.1. *A. communis* Methanol Extract (ACM) and Its Preparation of Topical Administration

The heartwood of *A. communis* was obtained from the Tainan district agricultural research and extension station, Council of Agriculture, Taiwan. The heartwood was cut into slices (2 kg) and macerated in 20 L methanol for one week with triplication. The methanol extract was concentrated by rotary vacuum evaporation and lyophilized with a freeze-dryer. The powders of the methanol extract of *A. communis* (ACM) were collected and stored in a moisture-proof container until used. Zero-point-five g and 0.25 g of ACM, respectively, were dissolved in 15 mL of 95% ethanol and then added into 25 mL polyethylene alcohol 400 (PEG400) and 10 mL of sterile water for preparing 1% and 0.5% of ACM external administration. 10 mL of 95% ethanol and 40 mL of PEG400 mixture solution was used as the vehicle group to compare the effect of ACM.

### 3.2. UV Absorption Spectrum of ACM

The UVA-UVB absorption analysis was performed for ACM. One mg of ACM was dissolved in methanol and diluted to 0.05 and 0.1 mg/mL. Samples were scanned by UV absorption at 260–400 nm using a UV-Vis spectrophotometer (DU730, Beckman Coulter Inc., Brea, CA, USA).

### 3.3. Administration of ACM on UVB Irradiation-Induced Skin Damage

Eight-week-old male hairless mice (Balb/c Nude), weighing 25–30 g, were purchased from the National Laboratory Animal Center, Taiwan. All animals were housed four per cage in an air-conditioned room with temperature maintained at 25 ± 1 °C and humidity at 55% ± 5% under a regular 12:12 h light/dark cycle during this experiment. All animals were fed the standard rodent chow and water *ad libitum* and received humane care in accordance to the “Guide for the Care and Use of Laboratory Animals” (National Academies Press, Washington, DC, USA, 1996). The method of UVB irradiation-induced skin damage followed that of Kim *et al.* with some modification [38]. Mice were randomly divided into four groups of four mice each. Group 1 was given the vehicle solution and served as a normal control without UVB irradiation. Group 2 was given the vehicle solution with UVB irradiation and served as a UVB-induced skin damage control. Group 3 and 4 were respectively treated with 1% and 0.5% ACM with UVB irradiation and served as the experimental group. The UVB source (312 nm) of simulated solar irradiation was provided by CL-1000M UV Crosslinker (UVP, Upland, CA, USA). The hairless mice of the UVB-induced group and the ACM administration group were exposed to UVB irradiation three times a week for 3 weeks with a total energy dose of 600 mJ/cm^2^ and two minutes for each time. The energy was set at 50 mJ/cm^2^ for the first week, and 75 mJ/cm^2^ was the exposure in the remnant 2 weeks. 50 μL for all groups were topically applied 30 min before UVB irradiation of the dorsal surface. All animal were sacrificed two days after the last UVB irradiation, and the skin morphology of all animals were photograph with a digital camera. The dorsal skin was immediately taken out and washed with ice-cold saline and stored at −80 °C. The skin samples were assessed for their histological, antioxidant and anti-inflammatory activities.

### 3.4. Histopathological Observation of the Skin

The mice skin tissues were fixed with 10% formalin buffer solution for 24 h and dehydrated with a sequence of ethanol solution and embedded in paraffin. The serial sections were cut 5 μm thick and stained with hematoxylin-eosin and then observed for the changes of skin damage by photomicroscope, including epidermis thickness and sunburn cells.

### 3.5. Determination of ROS Content

The skin tissue of mice in each experimental group was homogenized with 150 mM Tris-HCl buffer (pH 7.2) using a Polytron homogenizer (Brinkman Kinematica, Switzerland) to prepare 20% (*w*/*v*) skin homogenate. The content of reactive oxygen species (ROS) in skin tissue was determined using the modified method of Ebaid *et al.* [39]. Briefly, 20 μL of skin homogenates was mixed well with 980 μL of phosphate buffer saline and then aspirated with 50 μL of solution and 50 μL of 100 μM H2DCF-DA added. The samples were immediately added into a 96-well microplate for incubation for 30 min at room temperature in the dark. The optical density of the samples was measured by a fluorescence reader (FLx 800, Bio-Tek Instruments Inc., Winooski, VT, USA) at an excitation wavelength of 485 nm and an emission wavelength of 530 nm.

### 3.6. Determination of Anti-Lipid Peroxidation

The anti-lipid peroxidation was determined by using the modified method of Ohkawa *et al.* [40]. Briefly, 40 μL of homogenate was place into 2 mL microtube and then sequentially added with 40 μL of 9.8% SDS, 300 μL of 20% acetic acid, 300 μL of 0.8% TBA and 120 μL of distilled-deionized water. All samples were heated at 95 °C for 1 h and then quickly transferred into an iced-water bath. After that, 1 ml of *n*-butanol was added and each sample mixed well and immediately centrifuged at 3000 rpm, 25 °C for 10 min. 200 μL of supernatant of each sample was added in a 96-well microplate and measured by a microplate reader (ìQuant, Bio-Tek Instruments Inc., Winooski, VT, USA) at 532 nm.

### 3.7. Determination of Inflammatory Cytokines

The inflammatory cytokine levels of the cytosolic fraction of each skin homogenate were determined by Filip *et al.* [41]. The commercial ELISA kits of TNF-α and IL-1β (Quantikine, R&D Systems, Minneapolis, MN, USA) were respectively used to detect the level of TNF-α and IL-1β of the cytosolic fraction of each skin tissue sample, according to the procedure instruction.

### 3.8. Analysis of Inflammatory Protein Expression by Western Blot

Skin tissues were lysed in protein extraction solution (iNtRON Biotechnology Co., Korea) and protein extracted according to the procedure instructions. 50 μg of protein of each sample was denatured and subjected to SDS–PAGE using a 12% running gel and then transferred to nitrocellulose membranes. Membranes were incubated with anti-COX2, anti-cPLA2, anti-iNOS, anti-VCAM-1 (Santa Cruz Biotechnology, Santa Cruz, CA, USA) and anti-β actin (Enzo Life Sciences, Plymouth Meeting, PA, USA) antibodies for 24 h, and then, membranes were incubated with anti-mouse or anti-rabbit horseradish peroxidase antibody for 1 h. The immunoreactive bands were developed by chemiluminescent substrates and photographed by a gel image system (Alpha Innotech Co., San Leandro, CA, USA). All determinations were performed in triplicate.

### 3.9. Statistical Analysis

All data were expressed as the mean ± standard deviation of the indicated number of experiments. Data were analyzed by the Student’s *t* test to calculate statistical significance using 2003 Microsoft software. *p* < 0.05 was considered significant.

## 4. Conclusions

The results of the present study were the first to establish that ACM effectively prevents UVB-irradiation induced skin damage by decreasing epidermis thickness, sunburn cells, oxidative stress and inflammation. Consequently, we suggest that ACM can be a photoprotective agent in therapeutic and/or cosmetic products due to its UV absorption, antioxidant and anti-inflammation activity. We also suggest that ACM may be applied in a clinical setting and warrants further study.
